# Epidemiological Characteristics of the Lumpy Skin Disease Outbreak in Nawalpur, Nepal, 2022

**DOI:** 10.1155/2024/2003313

**Published:** 2024-07-31

**Authors:** Sujeeta Pokharel Dhakal, Surendra Karki, Sarah Vandyk, Mukul Upadhyaya, Krishnaraj Pandey, Aashish Dhakal, Sith Premashthira

**Affiliations:** ^1^ Veterinary Standards and Drug Regulatory Laboratory, Kathmandu, Nepal; ^2^ Food and Agriculture Organisation (FAO) Emergency Centre for Transboundary Animal Diseases (ECTAD), Kathmandu, Nepal; ^3^ Food and Agriculture Organisation (FAO) Emergency Centre for Transboundary Animal Diseases (ECTAD), Bangkok, Thailand; ^4^ Veterinary Epidemiology Section Animal Disease Investigation and Control Division, Kathmandu, Nepal; ^5^ Foot and Mouth Disease and Transboundary Animal Diseases Investigation Laboratory, Kathmandu, Nepal; ^6^ Nepal Polytechnic Institute, Chitwan, Nepal; ^7^ Regional Field Epidemiology Training Program for Veterinarians, Bangkok, Thailand

## Abstract

Lumpy skin disease (LSD) is an economically important and notifiable transboundary viral disease of cattle and water buffalo, predominantly transmitted by arthropod vectors. In recent times, LSD has emerged as a notable concern in Nepal, with the first outbreak reported in June 2020, in Morang district. In 2022, outbreaks of LSD were observed in several districts with Nawalpur district being the hard-hit district. The objective of this study is to provide insights into the epidemiological characteristics of LSD, to identify potential sources and associated risk factors for LSD outbreak in Nawalpur, and its financial impact. The overall morbidity rate was observed to be 28.02% (*n* = 431/1,538) and the mortality rate was 3.06% (*n* = 47/1,538), resulting in a case fatality rate of 10.90% (*n* = 47/431). The predominant clinical symptoms were skin nodules, lameness, and decreased milk production in milking animals. Dry cattle, including pregnant cows and cattle heifers were the most affected. Univariable logistic regression analysis identified factors linked to disease outbreaks, such as importing animals from disease prevalent regions, sharing feed and water, herd size, and the presence of clinical signs in neighboring farms. Multivariable analysis highlighted the significance of neighboring farms having sick animals in resulting disease outbreaks. Because of the substantial economic impact due to LSD, it is imperative to implement effective control and preventive measures. These include animal movement control and quarantine, following biosecurity protocols during nearby outbreaks, and targeted vaccination of susceptible populations to prevent further disease spread.

## 1. Background

Lumpy skin disease (LSD), is a viral disease affecting cattle and water buffalo (*Bubalus bubalis*) caused by LSD virus (LSDV), which belongs to the *Poxviridae* family and *Capripoxvirus* genus. The virus is phylogenetically distinct but cross-reacts serologically with strains causing sheep pox and goat pox; which are under the same genus [1, 2]. The LSDV is nonzoonotic and is predominantly transmitted by arthropod vectors such as mosquitoes, flies, and ticks. LSDV transmission by direct contact is considered ineffective [1, 3]. The clinical manifestations of LSD include fever, the presence of nodular lesions on the skin, mucous membrane of respiratory and digestive tracts, enlargement of subscapular and precrural lymph nodes, lameness, a sharp drop in milk production among milking animals, lacrimation, nasal discharges, and salivation [1, 4]. The incubation period ranges from 4 to 14 days but can extend up to 28 days [5]. Morbidity rates vary between 2% and 45%, and the mortality rate is usually less than 10% [1].

The World Organization for Animal Health (WOAH) has listed LSD as a notifiable transboundary disease due to its substantial economic losses, including decreased productivity, diminished hide quality, slowed growth, infertility, and mortality. In addition to these, the disease's impact on global livestock and livestock products' trade, along with the costly measures required for its control and eradication, has serious financial consequences for affected countries [1, 6].

LSD was first reported in Zambia in 1929, and then spread to entire African countries, except for few countries such as Algeria, Libya, Tunisia, and Morocco. In 1989, the disease extended into the middle east Asian countries, where it was first observed in Israel [7]. Between 2012 and 2018, LSD reached southeastern Europe, the Balkans and the Caucasus as part of the Eurasian LSD epidemic [8]. LSD was reported in Asia and the Pacific in 2019, affecting northwest China and Bangladesh. Subsequently, it was reported in India in 2020 [9]. Given the increased risk of LSD incursion into Nepal, the Department of Livestock Services (DLS) had strengthened quarantine at the border and initiated surveillance in high-risk districts. Veterinarians and animal health workers were trained to facilitate field diagnosis and promote early reporting, with a focus on implementing strict biosecurity measures, disinfecting infected premises, and vector control. However, despite such efforts, the first LSD outbreak occurred in Nepal in June 2020, in Morang; a district in close proximity to Indian border [10, 11]. The disease spread to three other districts within a period of a month. The Central Veterinary Laboratory confirmed the samples to be positive for LSDV. Furthermore, sequence analysis of four selected LSDV genes revealed that the Nepal's LSDVs resemble to the Bangladesh and Indian isolates and the historic isolates from Kenya [12]. Subsequently, by 2021, the disease had spread to over 30 districts of Nepal.

In the Gandaki province, the first outbreak of LSD was observed in Kaski and Tanahu districts in July 2020. In the same year, few cases of the disease were also reported from Nawalpur. However, in 2022, the district was severely affected. LSD is a relatively new disease in Nepal, and there have been recurring outbreaks over the past 3 years. The abundance of disease vectors and the subtropical climatic conditions prevalent in the Terai region of Nepal has possibly provided a favorable environment for transmission of LSD.

Many of these outbreaks were not thoroughly investigated, resulting in limited available information regarding the epidemiology of LSD spread in Nepal. This investigation was conducted on the ongoing outbreak at Nawalpur district aiming to provide insights into the epidemiological characteristics of LSD outbreaks, identify potential sources of LSD introduction in the Nawalpur district, and assess the associated risk factors contributing to LSD outbreak in Nawalpur district.

## 2. Materials and Methods

### 2.1. Study Area

Nepal is a landlocked country situated in South Asia sharing its eastern, western, and southern border with India and northern border with China. Administratively, Nepal is divided into seven provinces, 77 districts, and 753 local levels. Among the seven provinces, Gandaki Province (consisting 11 districts) ranks second in having the highest number of commercial livestock farms (i.e., 1238 farms in total) with 595 cattle farms and 639 buffalo farms (including mixed farms) in addition to other small farms [13]. Recently, an outbreak of LSD was reported from Nawalpur district of Gandaki Province. The Nawalpur district has an area of 1,043.1 km^2^ (402.7 miles^2^), and it is divided into eight municipalities. The outbreak investigation was conducted in two municipalities, namely, Devchuli municipality and Gaindakot municipality, from where the cases were seen.

### 2.2. Study Population

The dairy animal industry in Nawalpur district consists of both small and commercial farms of cattle and buffalo. These farms are categorized based on thresholds for commercial livestock farm identification, considering factors like farm type and geographical location. The population of cattle and buffalo in Nawalpur district is 74,545 and 68,492, respectively [14]. According to the Veterinary Hospital and Livestock Service Expert Centre (VHLSEC) in Nawalpur, the population of cattle and buffalo in Devchuli municipality is estimated at 4,492 and 6,994, and in Gaidakot municipality, there are 9,823 cattle and 4,211 buffalo, comprising both local and cross breeds. Most of these animals are reared by stall feeding, with a small percentage raised through the free grazing systems. Animals are mostly artificially inseminated and regularly vaccinated against foot and mouth Disease. Vaccination against LSD has recently been approved by the Nepalese government; however, during the investigation period, animals were not yet vaccinated against LSD. Few cases of LSD were observed in both municipalities in the previous year 2021, but they were not documented. Animals showing disease symptoms were generally treated with topical medications (such as potassium permanganate and herbal spray), antipyretics, and antibiotics.

### 2.3. Case Definition

For the outbreak investigation, at first, the descriptive study was conducted for understanding the basic characteristics of the outbreak, following the case definition for descriptive study. Later, for identifying the cause and associated risk factors, an analytical study was conducted.

#### 2.3.1. Case Definition for Descriptive Studies

A dairy farm in Nawalpur district with at least one animal showing following symptoms:

1. Skin nodules of about 2–5 cm diameter size on entire body or
2. Swollen lymph nodes with swelling in limbs,
3. With decreased milk production, if milking during July–December 2022, were considered as a suspected case farm.

A dairy farm in Nawalpur district during July–December 2022 was considered a confirmed case if at least one animal in that herd was confirmed positive for LSD with ELISA or PCR tests by the Veterinary Laboratory, Pokhara.

#### 2.3.2. Case Definition for Analytical Studies

A dairy farm in Nawalpur district during July–December 2022 that met the definition of suspected case or confirmed case was defined as case farm whereas, a farm in the area that did not meet the definition of a case farm, which did not have suspected signs or was not confirmed to be a case during the same period was considered as a noncase farm.

Case farms were selected based on backward and forward tracing from index farm as well as using data provided by VHLSEC and local veterinary units. Control farms were selected matching with case with time and place. The Veterinary Laboratory, Gandaki province, conducted the laboratory diagnosis. Altogether, 15 farms were confirmed to be LSD positive.

### 2.4. Data Collection

The Central Veterinary Laboratory and Veterinary Epidemiology Section of the Department of Livestock Services, in Kathmandu, received reports regarding the outbreak of LSD in Nawalpur from the Veterinary Laboratory, Pokhara. In response to this information, an outbreak investigation was initiated in the affected municipalities, carried by a team lead by Dr. Sujeeta (as principal investigator). The team included active participation from technicians representing local veterinary offices and members of the Dairy Farm Association in two municipalities. At first, the index farms in Devchuli and Gaidakot municipalities were visited. Data were collected through face-to-face interviews conducted using a semistructured questionnaire, prepared by the investigation team. The questionnaire included the general information of the farm and owner, animal information (demographics, affected animals, disease symptoms, and the treatment and control measures applied), farm management, biosecurity measures applied, and economics related to reduced milk production in the farm. The questionnaire was validated by testing it on eight farms before using for the actual study. Additionally, some noncase farm data were collected by phone call interviews using the same questionnaire. Data were also gathered from field observation and records of Veterinary Laboratory, Pokhara, and VHLSEC, Nawalpur. A case–control study was conducted to analyze the risk factors associated with disease transmission. For the descriptive study, all the reported farms along with farms found infected during investigation was taken as the total sample. And, for analytical study, noncase farms were selected in the ratio of 1 : 1.5 (based on expert opinion and considering the limitation of time and budget)

### 2.5. Data Management and Analysis

Data collection was carried out using a questionnaire. The data were then transferred to MS Excel version 2016 for further processing and analysis. The morbidity, mortality, and case fatality of the disease were calculated based on the collected data. The pattern of the outbreak and geographical distribution of cases were visualized with a timeline depicting the onset of the disease and with the maps generated using QGIS version 3.22.5. For the risk factor analysis, specific variables were identified based on a literature review and expert opinions. These variables included “introduced of new animals,” “shared drinking water,” “shared feed,” “separated new animals,” “symptoms in neighboring farms,” and “use of vector control measures.” To measure the strength of association between these potential risk factors and LSD outbreak, both univariable and multivariable logistic regression analyses were conducted using R Studio version 2023.06.0 + 421. A cutoff value of *p* ≤ 0.2 was employed to select variables for inclusion in multivariable analysis.

## 3. Results

### 3.1. History of the Outbreak

The first clinical case of LSD was observed on a commercial cattle farm located within Devchuli municipality on 31 August 2022. Animals were exhibiting clinical signs, including swollen lymph nodes, skin nodules of 2–5 cm diameter on their entire body, and decreased milk production among the lactating animals. The case was first reported to the local veterinarian, who subsequently identified similar cases in two additional farms. Suspecting the disease, local veterinarian reported the situation to the VHLSEC, Nawalpur, on 20 September 2022. The skin lesion and serum samples were sent to the Regional Veterinary Laboratory in Pokhara. The samples were tested with antibody ELISA and later confirmed positive with real-time PCR test.

### 3.2. Index Farm Information

**Figure 1:**
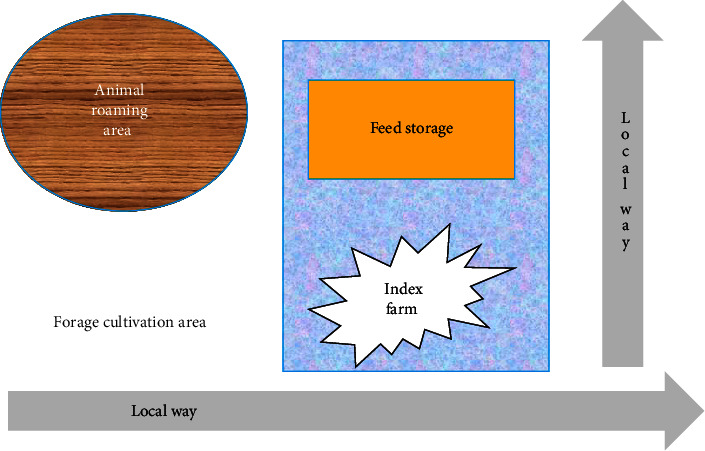
Layout of index farm.

The index farm was situated ~600 m away from the Mahendra Highway (East–West Highway) and was adjacent to a local road (Figure 1). The farm was enclosed with mesh wire fence. Animals were primarily stall-fed, sharing common feed trough and water trough. During the daytime, the cattle were allowed to roam and were kept outside the sheds within a confined area. There were cultivated forage surrounding the farm, and vectors including mosquitoes, houseflies, tabanus flies, and ticks were abundant during April–September. Ivermectin was used for the control of external and internal parasites. Animals were fed with hay and concentrates. The vehicles carrying feed and other supplies were not allowed to enter the farm.

**Figure 2:**
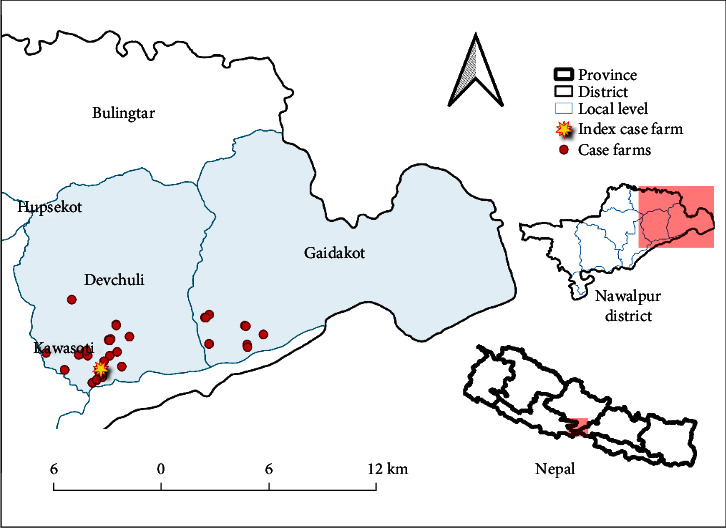
Index and case farms location in outbreak area.

Animals in the index farm were artificially inseminated by a local technician about a month before the onset of symptoms. Furthermore, the owner had recently sold a cow to a nearby farm one week prior to the manifestation of LSD-related symptoms on the farm. The onset of symptoms related to LSD was reported on the second infected farm on 6 October 2022. This farm was located within the same ward as the index farm. The disease symptoms were observed on 1 September 2022. Subsequently, farms near the index farm exhibited similar symptoms, between late September and early October (Figure 2). Despite the denials from local farmers regarding acquisition of new animals from neighboring district or boarder areas, history of animal movements was found in few farms during the investigation. A farm located 1.3 km away from the index farm displayed similar symptoms on 25 August 2022. This farm (which is a primary case in this study), also had a history of artificial insemination conducted 4 days prior to onset of LSD symptoms and had recently administered vaccination against FMD about 15 days before the onset of symptoms. Furthermore, farms near to the primary case farms were found to have introduced new animals from border regions.

### 3.3. Descriptive Study

A total of 87 dairy farms were investigated for the LSD outbreak in Devchuli and Gaidakot municipality. Among these, 36 farms had LSD cases occurring between 25 August 2022 and 5 December 2022. Figure 2 illustrates the geographical distribution of the index farm and the farms affected by LSD. The primary case farm was situated at 1.3 km from the index farm.

The initial case of LSD in the area was observed within Devchuli municipality. Additionally, the total number of cases, morbidity, mortality, and case fatality rates were higher in Devchuli in comparison to Gaidakot municipality (Table 1).

The affected animals were grouped based on their production stages, which included milking, dry (including pregnant), heifers, calves, and male cattle and buffalo. From the outbreak investigation (as shown in Table 2), it was found that the overall morbidity rate of the disease was 28.02% with the highest percentage of cases in dry cattle (47.62%). This was followed by cattle calves (33.33%) and heifers (32.73%). The overall mortality caused by the disease was 3.06%. The higher mortality rates were evident in cattle heifers (5.09%), followed by milking cattle (4.04%). Similarly, the overall case fatality was 10.9% with higher case fatality in heifers (15.56%) and milking cattle (12.95%).

### 3.4. Epidemic Curve of Case Farms

**Figure 3:**
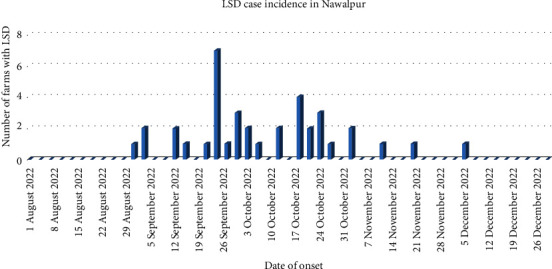
Epidemic curve of LSD outbreak in dairy farms, Nawalpur, Nepal.

The initial case reported (i.e., index case), was observed on 31 August 2022, in Devchuli. However, on tracing backwards from the index case, the primary case was found to have occurred on 25 August 2022. In Gaidakot, the first case was observed on 10 September 2022. The epidemic curve in Figure 3 reveals, that the highest number of LSD outbreaks were during September, with 16 farms affected, followed by 15 farms reporting LSD outbreaks in October.

### 3.5. Clinical Signs in Case Farms

**Figure 4:**
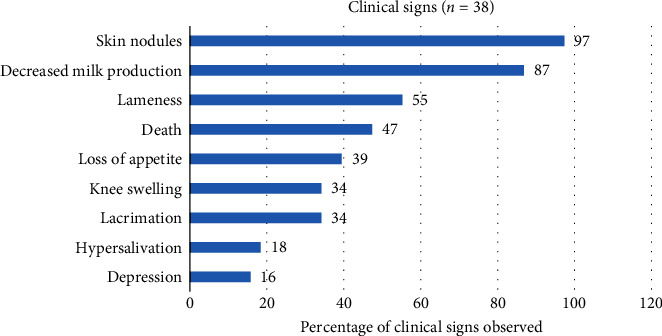
Percentage of case farms (*n* = 38) with specified clinical signs of LSD.

All farms affected by LSD outbreak exhibited one or more clinical symptoms of the disease. The major clinical signs observed included the presence of nodules in the skin, a reduction in milk production, and lameness in 97%, 87%, and 55% of the case farms, respectively (Figure 4).

### 3.6. Probable Risk Factors

**Figure 5:**
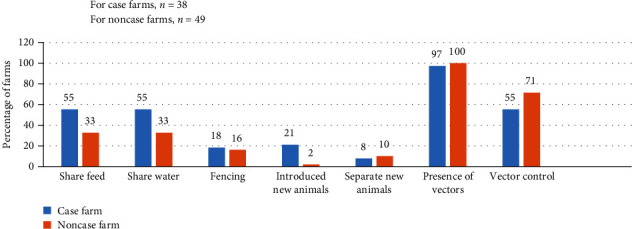
Probable risk factors for LSD outbreak.

From the data collected, it was found that 55% (*n* = 21/38) of case farms had a shared feeding trough and water trough. Additionally, 21% (*n* = 8/38) of the case farms had a recent history of introducing new animals into their herds, and 8% (*n* = 3/38) farms separated new animals before mixing to the herd. Furthermore, 55% (*n* = 21/38) of the case farms used vector control measures. A higher percentage of case farms (29%, *n* = 11/38) were found to use disinfectants compared to noncase farms (8%, *n* = 4/49). However, most of the farms commenced the use of disinfectants after the occurrence of the disease in their herds. Interestingly, a higher percentage of case farms (18%, *n* = 7/38) had fencing in their premises in comparison to noncase farms (16%, *n* = 8/49) as shown in Figure 5.

### 3.7. Analytical Statistics

#### 3.7.1. Univariable Analysis of Risk Factors

Nine variables were identified as probable risk factors for the outbreak. These variables included “herd size,” “introduced new animals,” “separate new animals,” “share feed,” “share water,” “symptoms in neighbor farms,” “presence of vectors,” “use of vector control,” and “fencing.” These variables were subjected to univariate analysis and the variables which had *p* value < 0.2 were considered the candidate variable for multivariable logistic regression (Table 3).

#### 3.7.2. Multivariable Analysis of Risk Factors

Out of six variables from univariable logistic regression which had *p* value < 0.2, only one variable was found to be significant in the final model, as shown in Table 4.

## 4. Discussions

In this study, the overall morbidity of the disease was 28.02%, the mortality was 3.06%, and case fatality was 10.90% which was alike the findings from Punyapornwithaya et al. [15] in Thailand, where the morbidity and mortality of LSD was observed to be 31% and 0.9%, respectively. Likewise, Abutarbush et al. [16] in Jordan, reported an average morbidity rate of 35.1% and a mortality rate of 1.3%, Khalil et al. [17] observed rates of 21% and 1% for morbidity and mortality, respectively, in Bangladesh. In contrast, Kasem et al. [18] reported a lower morbidity rate of 6% and a mortality rate of 0.99% for LSD in Saudi Arabia, and Sudhakar et al. [9] reported an apparent morbidity rate of 7.1% (0.75%–38.34%) with no recorded mortality within five different districts of Odisha in India, due to LSD. Weiss [19] stated that such variation in morbidity rate of LSD as a natural resistance to infection, which is not associated with immunity, occurs in ~50% or more of all cattle exposed to the natural or experimental infection. Additionally, he also mentioned a potential “life-long” immunity to LSD on animals recovered from apparent or inapparent natural infections, regarding to the presence of the neutralizing antibodies for at least 5 years. Weiss [19] also questioned the validity of observations of recurrent attacks of lumpy skin disease in the same animals by Huston [20], as infection by other unrelated virus “Alberton” also causes similar clinical syndrome and was not identified by the time.

During this outbreak investigation, it was noted that an infection occurred on a farm (referred to as the primary case farm) prior to the index farm. The primary case farm's proximity to the Indian border, where animals were sourced from local markets for sale in the domestic market, indicated that the movement of animals from this border area might be a potential source of infection for this outbreak. Moreover, the exchange of animals between farms within the affected region during the LSD outbreak period was identified. Statistical analysis revealed a significant association between disease occurrence and the presence of symptoms in neighboring farms. This highlights the potential role of animal movement in disease transmission.

From this investigation, the morbidity in dry cattle (including pregnant) was found higher followed by cattle calves, cattle heifers, and milking cattle. The mortality and case fatality rates were observed to be higher in cattle heifers and milking cattle. The mortalities were linked with LSD based on the severity and the characteristic signs and lesions of the disease that the animals had shown. Ahmed and Zaher [21] and Khalil et al. [17] reported similar finding of higher disease incidence in pregnant and young cows compared to those of moderate age.

Alhough the herd size, introduction of new animals, and sharing of feed and water were found to be associated with disease outbreak in the univariable analysis, their impacts were overshadowed by the presence of symptoms in neighboring farms during the multivariable analysis. Magori-Cohen et al. [3] and Paslaru et al. [22] emphasized that direct contact has a minor effect on virus spread but plays significant role in by mechanical transmission by flying biting insects. Vectors such as mosquitoes, houseflies, tabanus flies, and ticks were found in almost every farm in the outbreak area. Coetzer et al. [23], stated that factors such as inferior immune status and the presence of arthropod vectors are major predisposing factors for LSD outbreaks. Similarly, Kiplagat et al. [24] reported that the disease is spread by biting flies, direct transmission from the initial case, and the introduction of cattle from other parts of country in the case of sedentary herds in Kenya. Sprygin et al. [25] suggested that synanthropic house fly, *Musca domestica*, may also play a role in LSDV transmission, however this has not been clinically tested. Şevik and Doğan [8] stated that movement of animals prior to the major festive seasons are closely associated with the spread of LSD.

LSD causes a great economic burden on dairy farms. Thus, it is imperative to implement effective measures for its control and prevention. Our study has revealed a significant association between the presence of symptoms in neighboring farms and disease outbreak. Hence, for the control of the disease spread, it is important to prevent the entry of infected animal to the naive area and to control the vectors, which are the primary source of LSD transmission. One of the efficient ways to control the disease is to regulate the movement of affected animals. In this case, the dairy farmer association itself implied the control of purchase and sales of animals during the time of disease outbreak. Together with this, local authorities, recognizing the profound economic impact of the disease, should enforce stringent oversight. In Nepal, the internal quarantine check posts play a crucial role for managing the movement of the livestock from the LSD-affected zones. Additionally, the government mandates a 21-day quarantine for the animals suspected of LSD.

Early detection of the disease allows enough time for the preparation and implementation of the control measures. To achieve this, an efficient laboratory surveillance in districts at higher risk of LSD outbreak is crucial. The biosecurity measures are imperative to mitigate the spread of disease from nearby farms. Awareness must be given to the farmers, school students, and local technicians about the importance of adherence to the biosecurity protocols and how even a small negligence can create a loophole for the outbreak of such a costly disease. Along with this, a practical vector control strategy based on vector biology should be implied. For instance, for the control of mosquitoes, reducing the breeding sites of the insect by removing the standing water near the farms, using larvicides or larva eating fish in water bodies. Insecticides can be used for controlling the adult insects. Farm netting can also be considered as an option for reducing animal's exposure to insects. Together with these measures, educating the community about vector biology, disease transmission, and preventive measures is a must. Furthermore, maintaining farm cleanliness and regular disinfection are vital for disease prevention. Vaccination to the susceptible population with a vaccine coverage of more than 80% is needed to prevent further spread of the disease.

## 5. Conclusions

The morbidity of LSD was found to be less than 30% with a low mortality rate. Presence of skin nodules was the most predominant clinical signs along with decreased milk production, lameness, and death. A significant association was found between disease outbreak and presence of symptoms in nearby farm. The movement of animals between farms was found to spread the infection among farms and presence of vectors in farm acted as a predisposing factor for disease outbreak. LSD caused a significant impact to the farmers mostly due to the various direct and indirect losses.

## Figures and Tables

**Table 1:** Distribution of LSD outbreak in Nawalpur district.

Municipalities	Number of animals	Number of sick animals	Number of dead animals	Morbidity (%)	Mortality (%)	Case fatality (%)
Devchuli	983	367	43	37.33	4.37	11.72
Gaidakot	555	64	4	11.53	0.72	6.25

**Table 2:** Morbidity, mortality, and case -fatality of LSD in Nawalpur.

Animal group	Number of animals	Number of sick animals	Number of dead animals	Morbidity (%)	Mortality (%)	Case fatality (%)
Milking cattle	717	224	29	31.24	4.04	12.95
Dry cattle	189	90	3	47.62	1.59	3.33
Cattle heifer	275	90	14	32.73	5.09	15.56
Cattle calf	57	19	1	33.33	1.75	5.26
Milking buffalo	170	6	0	3.53	0.00	0.00
Dry buffalo	22	1	0	4.55	0.00	0.00
Buffalo heifer	62	0	0	0.00	0.00	0.00
Buffalo calf	36	1	0	2.78	0.00	0.00
Male	10	0	0	0.00	0.00	0.00
Total	1538	431	47	28.02	3.06	10.90

**Table 3:** Univariable logistic regression analysis.

Variables	Category	Cases (*n* = 38)	Noncases (*n* = 49)	OR (95% CI)	*p* values
Herd size	≥15 heads	22	15	3.12 (1.29–7.55)	0.012 ^*∗*^
	<15 heads	16	34	—	—
Introduced new animals	Yes	8	1	12.77 (1.52–107.10)	0.019 ^*∗*^
	No	30	48	—	—
Separate new animals	Yes	3	5	0.75 (0.17–3.38)	0.712
	No	35	44	—	—
Share feed	Yes	21	16	2.55 (1.06–6.11)	0.036 ^*∗*^
	No	17	33	—	—
Share water	Yes	21	16	2.55 (1.06–6.11)	0.036 ^*∗*^
	No	17	33	—	—
Symptoms in neighboring farms	Yes	36	11	62.09 (12.88–299.47)	<0.001 ^*∗*^
	No	2	38	—	—
Use of vector control	Yes	21	35	0.49 (0.20–1.20)	0.121 ^*∗*^
	No	17	14	—	—
Fencing	Yes	7	8	1.16 (0.38–3.54)	0.797
	No	31	41	—	—

*Note*.  ^*∗*^Represents the values considered for further analysis.

**Table 4:** Multivariable logistic regression analysis.

Variables	Odds ratio	95% CI	*p* value
Herd size	1.78	0.42–7.48	0.434
Introduced new animals	15.35	0.39–595.17	0.143
Use of vector control	3.29	0.71–15.17	0.126
Share feed	3.53	0.84–14.94	0.086
Symptoms in neighbor farms	88.45	13.27–589.36	<0.001 ^*∗*^

*Note*.  ^*∗*^Represents statistically significant value.

## Data Availability

The datasets used and/or analysed during the current study are available from the corresponding author on reasonable request.
